# Robotic-assisted total mesorectal excision with the single-docking technique for patients with rectal cancer

**DOI:** 10.1186/s12893-017-0315-x

**Published:** 2017-12-05

**Authors:** Ching-Wen Huang, Hsiang-Lin Tsai, Yung-Sung Yeh, Wei-Chih Su, Ming-Yii Huang, Chun-Ming Huang, Yu-Tang Chang, Jaw-Yuan Wang

**Affiliations:** 1 Graduate Institute of Medicine, College of Medicine, Kaohsiung Medical University, Kaohsiung, Taiwan; 2 Division of Colorectal Surgery, Department of Surgery, Kaohsiung Medical University Hospital, Kaohsiung Medical University, Kaohsiung, Taiwan; 3 Division of Trauma, Department of Surgery, Kaohsiung Medical University Hospital, Kaohsiung Medical University, Kaohsiung, Taiwan; 4 Division of General Surgery Medicine, Department of Surgery, Kaohsiung Medical University Hospital, Kaohsiung Medical University, Kaohsiung, Taiwan; 5 Department of Surgery, Faculty of Medicine, College of Medicine, Kaohsiung Medical University, Kaohsiung, Taiwan; 6 Graduate Institute of Clinical Medicine, College of Medicine, Kaohsiung Medical University, Kaohsiung, Taiwan; 7 Department of Radiation Oncology, Kaohsiung Medical University Hospital, Kaohsiung Medical University, Kaohsiung, Taiwan; 8 Division of Pediatric Surgery, Department of Surgery, Kaohsiung Medical University Hospital, Kaohsiung Medical University, Kaohsiung, Taiwan; 9 Center for Biomarkers and Biotech Drugs, Kaohsiung Medical University, Kaohsiung, Taiwan; 10 Center for Environmental Medicine, Kaohsiung Medical University, Kaohsiung, Taiwan; 11 Research Center for Natural products & Drug Development, Kaohsiung Medical University, Kaohsiung, Taiwan

**Keywords:** Robotic-assisted total mesorectal excision, Single-docking, Rectal cancer, R0 resection, Circumferential resection margin

## Abstract

**Background:**

The robotic system has advantages of high-definition three-dimensional vision and articular instruments with high dexterity, allowing more precise dissection in the deep and narrow pelvic cavity.

**Methods:**

We enrolled 95 patients with stage I-III rectal cancer (adenocarcinoma) who underwent totally robotic-assisted total mesorectal excision (TME) with single-docking technique at a single institution between September 2013 and December 2016.

**Results:**

Of the 95 patients, 48 (50.5%) and 30 (31.6%) patients had lower and middle rectal cancers, respectively. Of the 75 (78.9%) patients undergoing preoperative concurrent chemoradiotherapy (CCRT), 27 (28.4%) exhibited pathologic complete response (pCR). Only four (4.2%) patients underwent abdominoperineal resection and the sphincter preservation rate was 95.8%. R0 resection was performed in 92 (96.8%) patients. Circumferential resection margin (CRM) and distal resection margin (DRM) were positive in 2 (2.1%) and 1 (1.1%) patients, respectively. The anastomotic leakage rate was 5.4% (5/95 patients). The overall complication rate was 17.9% (17/95 patients); most of them were mild. No 30-day hospital mortality occurred, and no patients required conversion to open surgery. In 92 patients undergoing R0 resection, 2-year overall survival was 94% and 2-year disease-free survival was 83%.

**Conclusions:**

The results demonstrated that totally robotic-assisted TME with the single-docking technique is safe and feasible for patients with rectal cancer, with or without preoperative CCRT. Moreover, favorable pCR rate, R0 resection rate, CRM, DRM, sphincter preservation rate, and short-term oncological outcomes can be achieved by combining this approach with appropriate preoperative CCRT.

## Background

In the past three decades, several advancements including improvement in surgical techniques and the development of new therapeutic modalities have improved treatment outcomes of rectal cancers. Total mesorectal excision (TME) surgery, which was described by Heald and Ryall [1] in 1982, remarkably improves the clinical outcomes of patients with rectal cancer; thus it has served as the standard surgical procedure for such patients. A 5-year local recurrence rate of 5% in patients who undergone TME surgery alone was reported by MacFarlane et al. [2]. In addition, preoperative concurrent chemoradiotherapy (CCRT) considerably helps in improving the local recurrence rate in patients with locally advanced rectal cancer (LARC). A German study reported a considerable decrease in local recurrence in patients receiving preoperative CCRT [3, 4]. The similar results were also reported by other studies [5–7] and preoperative CCRT has been the recommended treatment for patients with LARC.

Laparoscopic rectal surgery with TME is still not accepted worldwide as the standard surgical procedure for rectal cancer treatment because it requires highly technically skilled surgeons experienced in minimally invasive surgeries [8, 9]. The robotic system (da Vinci® Surgical System, Intuitive Surgical, Inc., Sunnyvale, CA) has several advantages such as high-definition three-dimensional vision with up to 10× magnification, the articulatory instruments of the system, the surgeon-controlled camera platform, and stable traction provided by the robotic arm. Thus, dissection in the confined pelvic cavity can be performed more precisely by using this robotic system. Since the first robotic colon surgery in 2002 [10], the disadvantages of conventional laparoscopic colorectal surgery are expected to be solved by robotic systems. Several studies have reported that compared with conventional laparoscopic and open surgeries for rectal cancers, clinical and short-term oncological outcomes of robotic surgery are more favorable [11–14].

Rectal cancer surgery is a multiquadrant operation involving the left upper quadrant, left lower quadrant, and pelvic cavity. Surgical procedures include dissection of the lymph nodes; ligation of the inferior mesentery artery (IMA) and inferior mesentery vein (IMV); mobilization of the splenic flexure of the colon, descending colon, and sigmoid colon; and dissection of the pelvic. The hybrid technique with laparoscopic dissection of the lymph nodes, ligation of IMA and IMV, mobilization of the colon, and robotic dissection of the pelvic developed first. Thereafter, totally robotic surgeries with the dual-docking technique or single-docking flip-arm technique were performed. Several robotic surgical techniques including hybrid, totally robotic (including dual-docking and single-docking flip-arm techniques), and reverse hybrid are currently being used [15].

In the present study, we present a method of the single-docking technique without moving the robotic surgical cart and repositioning robotic arms to perform totally robotic radical rectal cancer surgery. In addition, we discuss the short-term oncological outcomes of patients with rectal cancer who underwent totally robotic-assisted TME with the single-docking technique.

## Methods

### Patients

We included 95 patients with stage I-III rectal cancer (adenocarcinoma) who underwent totally robotic-assisted TME with the single-docking technique with the da Vinci® surgical system at a single-institution between September 2013 and December 2016. This study was approved by the institutional review board of the Kaohsiung Medical University Hospital (KMUHIRB-E-20150003). Written informed consent to participate was obtained from each patient before performing the robotic surgery.

All patients routinely underwent preoperative colonoscopy and abdominal and pelvic computed tomography (CT) or magnetic resonance imaging (MRI) for preoperative staging. On the basis of the distance from the anal verge, rectal cancer was categorized into upper (11–15 cm), middle (6–10 cm), and lower (≤ 5 cm). Patients with T3, T4, or N+ rectal cancer received preoperative CCRT. Furthermore, the 5-fluorouracil, leucovorin, and oxaliplatin (FOLFOX) regimen was prescribed for patients with T4 or N+ rectal cancer and a fluoropyrimidine-based regimen was prescribed for patients with T3 N0 rectal cancer. Long-course radiotherapy (LCRT, total 5000 cGy in 25 fractions) was concurrently administered. Totally robotic-assisted TME with the single-docking technique was scheduled after more than 6 weeks after radiotherapy completion.

Clinicopathological features and perioperative parameters or outcomes were collected and evaluated, including age; sex; histological type; tumor, node, and metastasis (TNM classification); perineural invasion; vascular invasion; time interval between completion of preoperative radiotherapy and robotic surgery; tumor location (distance from anal verge); pre-CCRT, preoperative, and postoperative serum carcinoembryonic antigen (CEA) levels; and body mass index (BMI). The TNM classification was defined according to the criteria of the American Joint Commission on Cancer (AJCC)/International Union Against Cancer (UICC) [16]. The tumor regression grade (TRG) was evaluated according to the AJCC system [17]. Perioperative outcomes were collected and evaluated, including surgical procedures, docking time, console time, operation time, estimated blood loss, time of the first flatus passage, time of resuming soft diet, duration of postoperative hospital stay, and postoperative first day visual analog scale (VAS) pain score.

Patients were regularly followed up, including the collection of their clinical outcomes and survival statuses. History-taking and physical examinations were performed postoperatively every 3 months during the first 2 years and then every 6 months during the following 3 years. Measure of serum CEA levels were performed every 2–3 months postoperatively. A colonoscopy was performed approximately 1 year after surgery. Repeat colonoscopy was typically recommended at 3 years, unless follow-up colonoscopy indicated advanced adenoma (villous polyp, polyp > 1 cm, or high-grade dysplasia). Abdominal and pelvic CT scans were annually performed during postoperative 3 years in patients with stage II–III disease.

### Surgical procedure

**Fig. 1:**
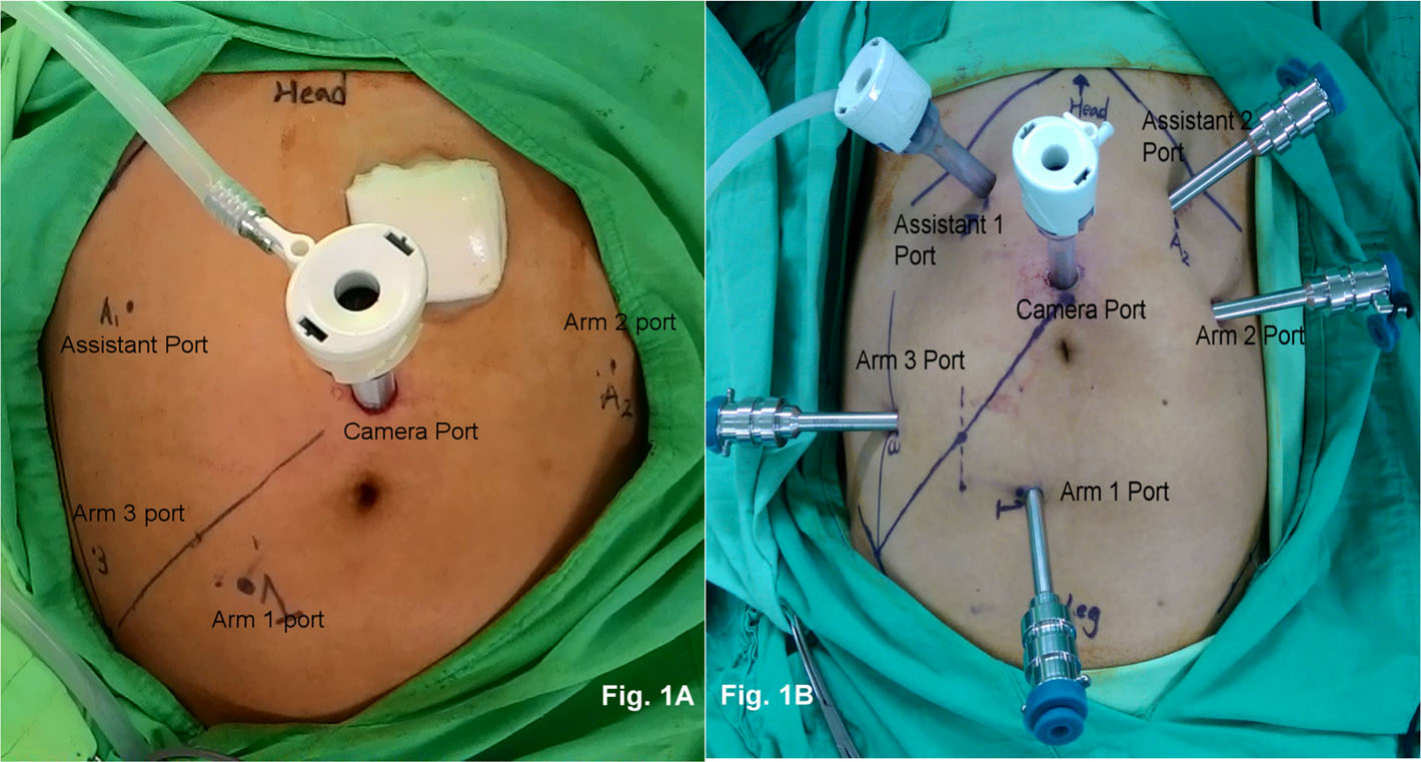
**a** Port positions during single docking with the five-port technique. **b** Port positions during single docking with the six-port technique

For all patients, we performed laparoscopic examinations to initially examine the intra-abdominal cavity. If an adhesion was observed, we performed laparoscopic lysis. Subsequently, we performed robotic surgery. The single-docking technique with five or six ports (Fig. 1) was used as the docking method, as described in our previous studies [18, 19]. One 12-mm camera port was placed 2 cm superior to the umbilicus. One 8-mm port (Arm 1 port) was inserted approximately 2 cm inferior to the line between the location of the camera port down to the right anterior superior iliac spine and slightly medial to the right mid-clavicular line (MCL). One 8-mm port (Arm 3 port) was inserted right laterally 8 cm from the Arm 1 port. One 12-mm port (assistant port) was inserted at the right MCL, approximately 4 cm inferior to the right costal margin. One 8-mm port (Arm 2 port) was inserted left laterally 8 cm from the camera port. One 12-mm port (assistant port) was inserted at the left MCL, approximately 2–4 cm inferior to the left costal margin. A monopolar permanent cautery spatula (Intuitive Surgical) was used in Arm 1, a Maryland bipolar forceps (Intuitive Surgical) was used in Arm 2, and a double fenestrated grasper (Intuitive Surgical) was used in Arm 3. The da Vinci® Si Surgical System was docked over the left flank of a patient. We performed medial to lateral dissection. Peritoneal incision at the level of the sacral promontory was performed first. The dissection was extended downward and then upward to the root of the IMA. We performed so-called high dissection and low ligation [19] in the form of D3 lymph node dissection and low-tie ligation of the IMA by using endo clips (Hem-O-Lok, Weck Closure Systems, NC) with preservation of the left colic artery in all patients. The inferior mesenteric vein (IMV) was also recognized, but was not ligated and divided instantly. If there was tension during the colonic anastomosis, the IMV would be ligated by using endo clips (Hem-O-Lok, Weck Closure Systems, NC) and divided. The splenic flexure of the colon was not routinely mobilized, if its mobilization was dependent on the tension of the anastomosis. Totally robotic-assisted TME with single-docking technique was performed in all patients.

After the sigmoid or descending colon, mesocolon, entire rectum and mesorectum were mobilized completely, low anterior resection (LAR) with the double-stapled technique, intersphincteric resection (ISR) with coloanal anastomosis and loop colostomy, or abdominoperineal resection (APR) was accordingly performed [18, 19]., LAR with the double-stapled technique was used for a tumor located in the upper and mid rectum. The rectum was divided by the assistant using an Endo GIA stapler (Endo GIA™ Reinforced Reload with Tri-Staple™ Technology, Medtronic) or ECHELON FLEX™ Powered ENDOPATH® stapler (Ethicon US, LLC) with one to three 60-mm reloads before the da Vinci® Si Surgical System was undocked. We extracted the specimen through the extended camera port wound with the Alexis® wound proctor and resected it. We then re-established the pneumoperitoneum and performed laparoscopic anastomosis by using a circular EEA stapler. Intraoperative dye test [20] was routinely performed to examine potential anastomotic leakage after LAR using the double-stapled technique. For a tumor located in the low rectum, ISR with coloanal anastomosis and loop colostomy was used. We used the Lone Star Retractor System® (Lone Star Medical Products Inc., Houston, TX) for ISR and subsequently we extracted the specimen and resected it transanally (natural orifice specimen extraction). Coloanal anastomosis was performed using the hand-sewn method. A protective loop colostomy of transverse colon was performed. Finally, we checked for bleeding in the abdominal cavity by using the traditional laparoscope and placed a drain tube in the pelvic cavity.

### Statistical analysis

We used the Statistical Package for Social Sciences, Version 19.0 (SPSS Inc., Chicago, IL) to statistically analyze all data. All patients were followed up until their death, last follow-up, or December 31 2016. The time required to position the robot and secure the robotic arms to the corresponding port sites was defined as the docking time. The total time during which the surgeon performed any procedure by using the robotic system was defined as the console time. The time between the initial skin incision and wound closure completion was defined as the operation time. We analyzed the learning curves indicated by various console and operation times by using a seven-case simple moving average method. A *P* value of < 0.05 denoted statistical significance. Overall survival (OS) was defined as the time from the date of primary treatment to the date of death from any cause or the date of last follow-up. Disease-free survival (DFS) was defined as the time from the date of primary treatment to the date of diagnosis of recurrence or metastatic disease or the date of last follow-up. OS and DFS were calculated by using the Kaplan–Meier method.

## Results

### Patients’ characteristics and perioperative outcomes

The baseline characteristics and perioperative outcomes of 95 patients with rectal cancer who underwent totally robotic-assisted TME with the single-docking technique were summarized in Table 1. The median age and BMI of the patients was 62 (range, 28–88) years and 23.54 (range, 17.20–34.02) kg/m^2^, respectively. Of the 95 patients, 48 (50.5%), 30 (31.6%), 17 (17.9%) had lower, middle, and upper rectal cancers, respectively. The median distance of the tumor from the anal verge was 5.5 (range, 1.0–15.0) cm.

**Table 1:** Baseline characteristics and perioperative outcomes of 95 patients with stage 0-III rectal cancer undergoing robotic-assisted total mesorectal excision

Characteristic	
Age (years, median) (range)	62 (28–88)
Gender	
Female	35 (36.8%)
Male	60 (63.2%)
Tumor distance from anal verge (cm)	
≦ 5 (Lower)	48 (50.5%)
6–10 (Middle)	30 (31.6%)
11–15 (Upper)	17 (17.9%)
Distance from anal verge (cm, median) (range)	5.5 (1–15)
Pre-operation CCRT	
Yes	75 (78.9%)
No	20 (21.1%)
Pre-operation chemotherapy regimen	75
FOLFOX	58 (77.3%)
Fluoropyrimidine-based	17 (22.7%)
Time interval between radiotherapy completion and robotic surgery (day, median) (range) (75 patients undergoing pre-operation chemotherapy)	82 (41–203)
ASA classification	
II	52 (54.7%)
III	43 (45.3%)
BMI kg/m^2^ (Median) (range)	23.54 (17.20–34.02)
Procedure	
LAR	59 (62.1%)
ISR	32 (33.7%) (including 3 transabdominal ISR)
APR	4 (4.2%)
Protective Diverting Colostomy	
Yes	38 (40.0%)
No	57 (60.0%)
Docking Time (min, median) (range)	5 (3–22)
Console Time (min, median) (range)	200 (130–435)
Operation Time (min, median) (range)	325 (210–795)
Estimated blood loss (mL, Median)	80 (15–1050)
Time of first flatus passage (day) (Median, range)	2 (1–10)
Time of resuming soft diet (day) (Median, range)	4 (2–15)
Postoperative hospital stay (day) (Median, range)	6 (5–32)
Postoperative first day VAS pain score (Median, range)	3 (1–8)

*APR* abdominoperineal resection, *AR* anterior resection, *ASA* American Society of Anesthesiologists, *BMI* Body mass index, *CCRT* Concurrent chemoradiotherapy, *ISR*, intersphenteric resection, *LAR* low anterior resection, *VAS* visual analog scale

The most frequent surgical procedure was LAR (59/95, 62.1%). ISR with coloanal anastomosis was performed in 32 (33.7%) patients, and APR was performed in 4 (4.2%) patients. Moreover, of the 32 patients undergoing ISR, 3 underwent transabdominal ISR and their tumor distances from the anal verge were 2–4 cm. Positive dye leakage after the completion of anastomosis was identified in six patients who had undergone LAR. Protective colostomies were performed accordingly. Finally, protective diverting loop transverse colostomy was performed in 38 (43.9%) patients, including 32 patients and 6 patients who underwent ISR and LAR, respectively. Sphincter preservation rate was 95.8%. The median estimated blood loss including tissue fluid after CCRT was 80 mL. The median time of the first flatus passage and resuming soft diet postoperatively was 2 and 4 days, respectively. The median duration of postoperative hospital stay was 6 days (range, 5–32).

### Postoperative complications

The postoperative complications are summarized in Table 2. Postoperative complications were observed in 14 patients with 17 episodes (17.9%). Three patients who developed intraabdominal abscess, CT-guided pigtail drainage were subsequently performed in 2 patients. Anastomosis leakage was observed in 5 (5.4%) patients who underwent LAR with the double-stapled technique, and loop colostomy of transverse colon was subsequently performed. Four (4.2%) patients developed stenosis of coloanal anastomosis and underwent dilation using a colonoscope. Urethral injury during ISR was noted in one (1.0%) patients. According to the Clavien-Dindo Classification, all post-operative ileus, urinary tract, and pulmonary complications were of grades I, and the patients recovered after conservative treatment. Moreover, no 30-day hospital mortality occurred.

**Table 2:** Postoperative complications in 95 patients with stage 0-III rectal cancer undergoing robotic-assisted total mesorectal excision

Complications	Number (%)	Management
Post-operative bleeding	1 (1.0%)	Laparotomy
Intra-abdominal infection/abscess	3 (3.2%)	2: conservative treatment
		1: CT-guided pig-tail drainage
Coloanal Anastomosis Stenosis	4 (4.2%)	Colonoscopic dilation
Ileus	1 (1.0%)	Conservative treatment
Anastomosis leakage	5 (5.4%)	Loop transverse colostomy
Urethral injury	1 (1.0%)	Conservative treatment
Pulmonary complication	2 (2.1%)	Conservative treatment
Total	17 (17.9%)	

### Pathological outcomes and oncological outcomes

The pathological characteristics and oncological outcomes of all 95 patients are listed in Table 3. Preoperative clinical staging demonstrated that the majority of the patients had locally advanced rectal cancers including T3 in 61 (64.2%) patients, T4 in 13 (13.7%) patients, or N+ in 57 (60.0%) patients. Therefore, preoperative CCRT was performed in 75 (78.9%) patients, including FOLFOX regimen in 58 (77.3%) patients with cT4 or cN+ disease, fluoropyrimidine-based regimen in 17 (22.7%) patients. The median number of harvested lymph nodes and apical lymph nodes was 9 (range, 0–36) and 2 (range, 0–15), respectively. However, positive apical lymph node metastasis was observed in only 3 (2.9%) patients. The median distance of the distal resection margin (DRM) and circumferential resection margin (CRM) was 2.30 and 1.0 cm, respectively. CRM and DRM were positive in 2 (2.1%) and 1 (1.1%) patients, respectively. R0 resection for primary rectal cancer was performed in 92 (96.8%) patients. Of the 75 patients who received preoperative CCRT, a pathologic complete response (pCR) of the primary tumor was observed in 27 (28.4%) patients. 28 (37.3%), 30 (40.0%), 11 (14.7%), and 6 (8.0%) patients exhibited complete response (TRG 0), moderate response (TRG 1), minimal response (TRG 2), and poor response (TRG 3), respectively. The median time interval between radiotherapy completion and robotic surgery was 82 (range, 41–203) days.

**Table 3:** Clinicopathologic characteristics and oncological outcomes of 95 patients with stage 0-III rectal cancer undergoing robotic-assisted total mesorectal excision

Preoperative clinical staging	
Tumor depth	
T1	2 (2.1%)
T2	19 (20.0%)
T3	61 (64.2%)
T4	13 (13.7%)
Lymph Node metastasis	
N0	38 (40.0%)
N1	40 (42.1%)
N2	17 (17.9%)
AJCC^a^ Stage (Clinical)	
I	14 (14.7%)
II	24 (25.3%)
III	57 (60.0%)
Postoperative pathological outcomes	
Histology	
Well differentiation	16 (16.9%)
Moderate differentiation	76 (80.0%)
Poor differentiation	3 (3.1%)
Tumor size	
< 5 cm	85 (89.5%)
≥ 5 cm	10 (10.5%)
Tumor size (cm, mean ± SD) (range)	2.46 ± 1.652 (0–8)
Tumor depth	
T0	29 (30.5%)
Tis	1 (1.0%)
T1	14 (14.7%)
T2	20 (21.1%)
T3	28 (29.5%)
T4	3 (3.2%)
Lymph Node metastasis	
N0	73 (77.1%)
N1	19 (19.8%)
N2	3 (3.1%)
AJCC Stage (Pathologic)	
0	27 (28.4%)
I	27 (28.4%)
II	19 (20.0%)
III	22 (23.2%)
Tumor Regression Grade (75 patients with preoperative CCRT)	
0	28 (37.3%)
1	30 (40.0%)
2	11 (14.7%)
3	6 (8.0%)
Harvested Lymph Node (median) (range)	9 (0–36)
Harvested Apical Node (median) (range)	2 (0–15)
Distance of distal resection margin (cm, median) (range)	2.30 (0.2–6.5)
Distance of circumferential resection margin (cm, median) (range)	1.0 (0.2–3.5)
Distal resection margin	
Free	94 (98.9%)
Positive	1 (1.1%)
Circumferential resection margin	
Free	93 (97.9%)
Positive	2 (2.1%)
Resection Degree of Primary tumor	
R0	92 (96.8%)
R1	3 (3.2%)
Oncological outcomes	
Follow-up periods (months, median) (range)	25.6 (6.6–52.2)
R0 resection	91
Locoregional recurrence	5 (5.5%)
Distant metastasis	10 (11.0%)
Liver + Lung	1 (1.1%)
Lung	5 (5.5%)
Liver	2 (2.2%)
Chest Wall	1 (1.1%)
Peritoneal carcinomatosis	1 (1.1%)
R1 resection	3
Local recurrence	1 (33.3%)
Lung	1 (33.3%)
Peritoneum	1 (33.3%)

^a^ *AJCC* American Joint Commission on Cancer

**Fig. 2:**
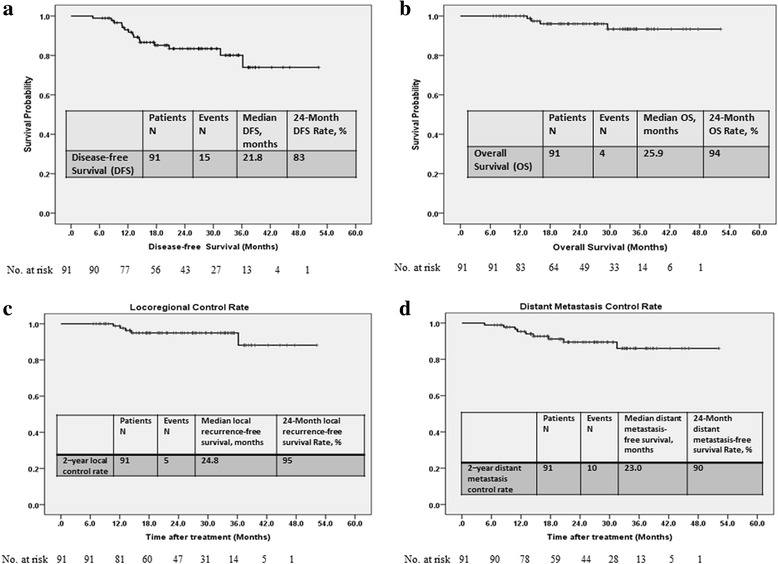
The Kaplan–Meier survival curves. **a** Disease-free survival. **b** Overall survival. **c** Locoreginal control rate. **d** Distant metastatsis control rate

The median follow-up duration of 95 patients from the primary treatment was 25.6 (range, 6.6–52.2) months. One patient undergoing local excision of primary tumor and radiotherapy at other hospital underwent chemotherapy with FOLFOX regiment and robotic ISR and coloanal anastomosis at our hospital after local recurrent tumor developed. We excluded this patient to analyze the oncological outcomes of the patients with undergoing R0 resection. Of 91 patients undergoing R0 resection, local recurrence and distant metastases were noted in 5 (5.5%) and 10 (11.0%) patients, respectively. At a median follow-up duration of 25.6 months, the 2-year OS was 94% and 2-year DFS was 83% (Fig. 2). Furthermore, 2-year local control rate and 2-year distant metastasis control rate were 95% and 90%, respectively.

### Learning curve of robotic CRC surgery

**Fig. 3:**
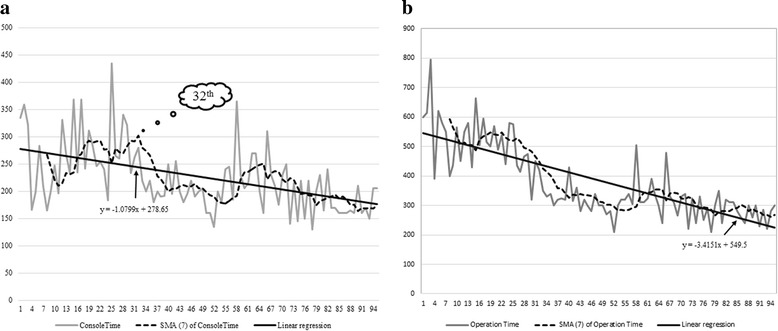
Learning curves for robotic rectal surgery. **a** Console time, all patients. **b** Operation time, all patients

The learning curves in terms of console and operation time are presented in Fig. 3. The median docking, console and operation time was 5 (range, 3–22), 200 (range, 130–435), and 325 (range, 210–795) minutes, respectively. A linear regression analysis indicated a decreasing trend for console time. The first plateau of console time was observed after 32 patients. The mean console time for the first 32 patient was significantly longer than that of the remaining patients (270.09 ± 64.830 vs 200.27 ± 42.080 min, *P* < 0.001). The linear regression analysis of operation time also indicated a decreasing trend for operation time. The mean operation time for the first 32 patient was significantly longer than that of the remaining patients (516.09 ± 11.460 vs 306.03 ± 6.804 min, *P* < 0.001).

## Discussion

In this study, we presented our experiences and short-term clinical and oncological outcomes of 95 patients with stage I-III rectal cancer who underwent totally robotic-assisted TME with the single-docking technique. The single-docking technique was performed in the complete procedure of totally robotic-assisted radical rectal cancer surgery without moving the robotic surgical cart and repositioning robotic arms. Meanwhile, we demonstrate that this technique is safe and feasible for patients with rectal cancer, with or without preoperative CCRT. Upmost important, favorable short-term clinical and oncological outcomes can be achieved by combining this approach with appropriate preoperative CCRT.

The hybrid technique was the first technique used in robotic rectal surgery, and many robotic rectal surgeries have been performed using the hybrid technique. However, with the hybrid technique, the advantages of the robotic system could not be utilized during the laparoscopic phase. The dual docking technique requires the movement of the robotic surgical cart and repositioning of robotic arms [21]. Hellan et al. first performed a robotic rectal surgery by using the hybrid technique [22] and then by using the single-docking technique [23]. Ahmed et al. [15] reported the experience and clinical outcomes of 100 patients who underwent robotic rectal surgery with the single-docking modified flip-arm technique. Luca et al. [24] used the single-docking technique to perform mobilization of the splenic flexure and TME. The surgical cart was not moved and the robotic arms were not repositioned during the surgery. The port sites of robotic arms used in this present study were different from those used in the study of Luca et al. [24].

In our study, the mean console time of the first 32 patients was significantly longer than that of the remaining patients. By using a standardized approach and more practice, robotic rectal surgery with TME can be performed safely and the console time can be reduced significantly. The results of this study were consistent with those of a meta-analysis conducted by Scarpinata et al. [25]. The selection criteria for robotic surgery in this meta-analysis were obesity, male sex, preoperative radiotherapy, and tumors in the lower two-thirds of the rectum. Though 78 (82.1%) patients had middle to low rectal cancers, the pCR was in 28.4% of patients and TRG 0 and 1 in 77.3% of patients. The pCR rate observed in our study (28.6%) is relatively higher than that reported in previous studies (10–30%, with less than 20% in most of studies) [26, 27]. The sphincter preservation rate achieved in our study was 96.1%, which is comparable with that reported by Kim et al. [28] and Saklani et al. [29] (Table 4).

**Table 4:** Comparison of clinical and perioperative outcomes by robotic-assisted TME^a^

Study	Country (year)	Operation type	Sample size	Lower rectum (%)	Preoperative CCRT^b^ (%)	Conversion Rate (%)	Estimated blood loss (mL)	Overall complications (%)	Anastomostic leakage (%)	Rate of sphincter preservation (%)	DRM^c^ (cm)	Positive CRM^d^ (%)
Present study (Huang et al.)	Taiwan (2017)	Totally robotic (single-docking)^g^	95([y]p Stage 0-III)	50.5	78.9	0	80 (15–1050)	17.69	5.4	95.8	2.3 (0.2–6.5.)	2.1
Baek et al. [11]	Korea (2011)	Hybrid	41 ([y]p Stage 0-III)	36.6^e^	80.5	7.3	200 (20–2000)	22.0	7.3	85.4	3.6 (0.4–10)	2.4
Park et al. [12]	Korea (2011)	Hybrid	52 ([y]p Stage 0-III)	60.4^f^	23.1	0	NA	19.2	9.6	100	2.8	1.9
Hellan et al. [14]	USA (2015)	Totally robotic or Hybrid	425 ([y]p Stage I-IV)	31.3	51.3	5.9	119 ± 164	40.2	8.7	NA	3.0 ± 2.0	0.9
Ahmed et al. [15]	UK (2016)	Totally robotic (single-docking)^h^	83	NA	21.7	0	10 (0–200)	49	2	88.0	2.7 (0.4–8.0)	3.6
Hellan et al. [22]	USA (2007)	Hybrid	39 ([y]p Stage I-IV)	53.9^f^	84.6	2.6	200 (25–6000)		12.1	84.6	2.65 (0.4–7.5)	0
Luca et al. [24]	Italy (2009)	Totally robotic (single-docking)^g^	28 ([y]p Stage I-IV)	NA	0	0	68 ± 138 (0–600)	NA	NA	75.0	2.5 ± 1.3 (0.6–5.5)	0
Kim et al. [28]	Korea (2016)	Totally robotic (single-docking)^h^	33 ([y]p Stage 0-III)	NA	100	6.1	232.0 ± 180.0	45.6	NA	93.9	2.2 ± 1.5	16.1
Saklani et al. [29]	Korea (2013)	Totally robotic (single-docking)^h^	74 ([y]p Stage 0-III)	NA	100	1.4	180 ± 28.1 (0–1100)	16.2	5.4	97.3	1.7 ± 1.4 (0.1–6.0)	4
Pai et al. [35]	USA (2015)	Dual docking or Hybrid	101 ([y]p Stage 0-IV)	28.7	74.3	4	190 ± 128	28.7	6.3	79.2	3.5 ± 2.7 (0.1–16.3)	5
Kim et al. [36]	Korea (2016)	Totally robotic (single-docking)^h^	60 ([y]p Stage 0-IV)	56.7^e^	36.7	0	74.2 ± 50	15	5	93.4	3.1 ± 1.7	11.7
Feroci et al. [37]	Italy (2016)	Totally robotic (single-docking)^g^	53 ([y]p Stage 0-III)	NA	49.1	3.8	60.8 (0–400)	26.4	5.7	100	2.5 (0.5–10)	0
Cho et al. [38]	Korea (2012)	Totally robotic (single-docking)^h^	278 ([y]p Stage 0-III)	24.8	32.7	0.4	179.0 ± 236.5	25.9	10.4	100	2.0 ± 1.4	5.0
Yamaguchi et al. [39]	Japan (2016)	Totally robotic (single-docking)^g^	203 ([y]p Stage 0-IV)	60.1^f^	0.5	0	15.4 ± 26.4	9	1.5	95.1	2.8 ± 1.9	NA
Park et al. [40]	Korea (2015)	Hybrid	133 ([y]p Stage I-III)	24.8	11.3	0	77.6 ± 153.2 (0–700)	19.7	4.5	100	2.75 ± 2.14 (1–14)	6.8
Ghezzi et al. [41]	Brazil/Italy (2014)	Totally robotic (single-docking)^g^	65 ([y]p Stage 0-III)	100^f^	72.3	1.5	0 (0–175)	41.5	7.1	86.2	2.7 (1.6–4.4)	0
Ramji et al. [42]	Canada (2016)	Hybrid	26	NA	58	12	296 ± 155	42	8	85	2.96 ± 2.05	0
Hara et al. [43]	Korea (2014)	Totally robotic (single-docking)^h^	200 ([y]p Stage 0-IV)	56.5	27.5	0	190 (0–1500)	38.5	9.5	93.5	1.8 (0–22.0)	1.5
Bail et al. [44]	Korea (2013)	Totally robotic (single-docking)^h^	370 ([y]p Stage 0-IV)	26.8	21.1	0.8	245.7 ± 222.1 (10.0–1300.0)	24.6	7.7	99.2	2.6 ± 1.4	6.9

*NA* not avaliable

^a^TME total mesorectal excision

^b^CCRT concurrent chemoradiotherapy

^c^CRM circumferential resection margin

^d^DRM distal resection margin

^e^< 7cm

^f^Extraperitoneal

^g^without moving both the robotic surgical cart and repositioning robotic arms

^h^without moving the robotic surgical cart, but repositioning robotic arms

TME completeness is a representative of the quality of rectal cancer surgery. The two crucial parameters of TME completeness are CRM involvement and DRM distance. Moreover, CRM involvement has been reported as a prognostic factor for local recurrence and survival [30–33]. In this study, the rate of CRM involvement was 2.1%, with a median distance of 1.0 cm, which is comparable with that reported in the previous studies (0–16.1%) (Table 4). Moreover, the rate of DRM involvement was 1.1% with a median distance of 2.3 cm, which is comparable to that reported in the previous studies (1.5–3.9 cm) (Table 4). R0 resection for primary rectal cancer was performed in 92 (96.8%) patients. Of the 91 patients with undergoing R0 resection, 5 (5.5%) developed local recurrence and 10 (11.0%) developed distant metastasis.

Although 82.1% of our patients had middle to low rectal cancers with a median distance of 5.5 cm from the anal verge and 63.2% of our patients were men, we did not mobilize the splenic flexure in most of our patients and still could perform precise dissection during TME procedure even using our single-docking technique. However, we still achieved a comparable distance of DMR and favorable negative rates of DRM and CRM. Protective diverting colostomy was performed in 40.0% of the patients undergoing sphincter preservation surgery; however, the anastomosis leakage rate in our study was comparable with that reported in the literature (Table 4).

The single-docking technique used in the present study is safe and feasible for treating patients with rectal cancer. However, some technical problems still exist. External collisions of the robotic arms usually occur. By using a standardized approach and through more practice, the positions of the robotic arms can be determined and external collisions can be avoided. We always encountered arm collisions when performing pelvic dissection. To reduce the occurrence of arm collisions, we used a monopolar permanent cautery spatula in Arm 3 and a double fenestrated grasper in Arm 1. Complete mobilization of the splenic flexure through our single-docking technique is difficult. When it was necessary to mobilize the splenic flexure, we reset the setting of the robotic arms (flip-arm techniques) to enable the surgeon to control different robotic arms rather than redocking the surgical cart. The single-docking technique with six ports (two assistant ports) is recommended for situations where performing pelvic dissection is difficult, such as for patients with mid and low rectal cancers, a high BMI, narrow pelvis, heavy mesorectum, or T4 lesions; women with huge uterine myomas; and patients who have responded poorly to neoadjuvant CCRT.

This study has some limitations that should be addressed. First, this is a single-institution retrospective study including only 95 patients. Second, the interval of follow-up was short, with 25.6 months of median follow-up duration; thus, only short-term (2 year) survival and oncological outcomes were reported. Nevertheless, 2–year OS (94%) and the 2–year DFS (83%) observed in our study were consistent with those reported in previous studies (Table 5). Furthermore, 2–year local control rate (95%) and 2–year distant metastasis control rate (90%) were consistent with those reported in previous studies (Table 5) [34]. Third, we did not evaluate the postoperative outcomes of urinary and sexual functions.

**Table 5:** Comparison of short-term oncological outcomes by robotic-assisted TME^a^

Study	Country (year)	Local recurrence (%)	Distant metastasis (%)	Disease-free survival	Overall survival
Present study (Huang et al.)	Taiwan (2017)	2.4	14.5	83.0% (2–year)	95.0% (2–year)
Pai et al. [35]	USA (2015)	4	17	79.2% (3–year)	90.1% (3–year)
Kim et al. [36]	Korea (2016)	1.9	26.4	72.8% (4–year)	87.7% (4–year)
Feroci et al. [37]	Italy (2016)	1.9	17	79.2% (3–year)	90.2% (3–year)
Cho et al. [38]	Korea (2012)	1.8	12.2	81.8% (5–year)	92.2% (5–year)
Park et al. [40]	Korea (2015)	2.3	12.0	81.9% (5–year)	92.8% (5–year)
Ghezzi et al. [41]	Brazil/Italy (2014)	3.2	18.5	73.2% (5–year)	85.2% (5–year)
Hara et al. [43]	Korea (2014)	4.5	10	81.7% (5–year)	92.0% (5–year)
Bail et al. [44]	Korea (2013)	3.6	17.6	79.2% (3–year)	93.1% (3–year)

^a^ *TME* total mesorectal excision

## Conclusions

With comparable short-term clinical outcomes, we demonstrate that this technique is safe and feasible for patients with rectal cancer, with or without preoperative CCRT. Moreover, favorable short-term oncological outcomes can be achieved by combining this approach with appropriate preoperative CCRT. However, long-term oncological outcomes should be further investigated by conducting studies having a longer follow-up duration.

## Abbreviations

AJCC: American Joint Commission on Cancer
APR: Abdominoperineal resection
BMI: Body mass index
CCRT: Concurrent chemoradiotherapy
CEA: Carcinoembryonic antigen
CRC: Colorectal cancer
CRM: Circumferential resection margin
CT: Computed tomography
DFS: Disease-free survival
DRM: Distal resection margin
IMA: Inferior mesentery artery
IMV: Inferior mesentery vein
ISR: Intersphincteric resection
LAR: Low anterior resection
LARC: Locally advanced rectal cancer
LCA: Left colic artery
LCRT: Long-course radiotherapy
MRI: Magnetic resonance imaging
OS: Overall survival
pCR: Pathologic complete response
SMA: Simple moving average
TME: Total mesorectal excision
TRG: Tumor regression grade
UICC: International Union Against Cancer
VAS: Visual analog scale
